# Seriation in Paleontological Data Using Markov Chain Monte Carlo Methods

**DOI:** 10.1371/journal.pcbi.0020006

**Published:** 2006-02-10

**Authors:** Kai Puolamäki, Mikael Fortelius, Heikki Mannila

**Affiliations:** 1 Laboratory of Computer and Information Science, Helsinki University of Technology, Espoo, Finland; 2 Department of Geology and Institute of Biotechnology, University of Helsinki, Helsinki, Finland; 3 HIIT Basic Research Unit, University of Helsinki and Helsinki University of Technology, Helsinki, Finland; Princeton University, United States of America

## Abstract

Given a collection of fossil sites with data about the taxa that occur in each site, the task in biochronology is to find good estimates for the ages or ordering of sites. We describe a full probabilistic model for fossil data. The parameters of the model are natural: the ordering of the sites, the origination and extinction times for each taxon, and the probabilities of different types of errors. We show that the posterior distributions of these parameters can be estimated reliably by using Markov chain Monte Carlo techniques. The posterior distributions of the model parameters can be used to answer many different questions about the data, including seriation (finding the best ordering of the sites) and outlier detection. We demonstrate the usefulness of the model and estimation method on synthetic data and on real data on large late Cenozoic mammals. As an example, for the sites with large number of occurrences of common genera, our methods give orderings, whose correlation with geochronologic ages is 0.95.

## Synopsis

Seriation, the task of temporal ordering of fossil occurrences by numerical methods, and correlation, the task of determining temporal equivalence, are fundamental problems in paleontology. With the increasing use of large databases of fossil occurrences in paleontological research, the need is increasing for seriation methods that can be used on data with limited or disparate age information. This paper describes a simple probabilistic model of site ordering and taxon occurrences. As there can be several parameter settings that have about equally good fit with the data, the authors use the Bayesian approach and Markov chain Monte Carlo methods to obtain a sample of parameter values describing the data. As an example, the method is applied to a dataset on Cenozoic mammals. The orderings produced by the method agree well with the orderings of the sites with known geochronologic ages.

## Introduction

Seriation, the task of temporal ordering of fossil occurrences by numerical methods, and correlation, the task of determining temporal equivalence, are fundamental problems in paleontology. Fossils have been used for both tasks since the very beginnings of modern paleontology [1,2]. However, the recent advent of large fossil databases [3] and the increased emphasis on quantitative analysis of biological patterns in deep time (e.g., [4–7]) has to some extent changed both the nature and the primary purpose of these activities. The rules and procedures of conventional paleontological seriation (biostratigraphy) [8,9] are not easy to apply in a satisfactory way to large datasets compiled from a wide variety of sources, often without associated data concerning the local distribution of fossil taxa in the rock sequences from which they derive. Conversely, occasional but valuable data regarding lithostratigraphic superposition, geochronologic age estimates, etc., are frequently available but difficult to apply in a global setting. The increasing use of large datasets in paleontological research implies a growing need for methods that do not only order the sites into a temporal sequence based on the distribution of taxon occurrences, but also can make use of a variety of other kinds of readily available stratigraphic information. This need is perhaps most acute in situations such as the continental record of the Eurasian Cenozoic (ca 23 million to 2 million years ago), where the majority of localities are from isolated quarries outside a rock-context and stratigraphic superposition therefore cannot be directly determined (e.g., [10,11]).

In the past several decades, both seriation and correlation of fossil occurrences by numerical methods have in fact become practically feasible alternatives to conventional biostratigraphy. The computational solutions that have been developed for correlation and seriation have much in common, but the implementations differ depending on the purpose and the nature of the data (e.g., the CHRONOS [12] and PAST [13,14] initiatives; also see [15]).

Here we are explicitly concerned with the task of seriation, for which methods based on several distinct approaches are available. These include the graph-theoretical unitary associations method by Guex et al. [16–18], parsimony analysis [19–22], and Bayesian methods [23]. John Alroy [5,6,24–27] has developed and applied techniques based on estimating taxon ranges and maximizing the fit of these hypothesized ranges to independent stratigraphic information, including known stratigraphic superposition of localities. ([5] gives the latest review of this approach.)

A fossil site (a collection of fossil remains collected from some location, typically in a sedimentary deposit) may be loosely regarded as a snapshot of the set of taxa that lived at a certain location at approximately the same time. Sites and their taxa may be described as an occurrence matrix, i.e., a 0–1 matrix, where the rows correspond to sites and the columns correspond to taxa: a one in entry (i,j) means that taxon j has been found at site i. The snapshot may capture a smaller or larger proportion of the taxa that were actually present, a smaller or larger area, and a shorter or longer time interval, and it may be biased in different ways. It is therefore clear that the ones and zeros in such a matrix are not all equal. Some presences will be weakly founded on single specimens, others on hundreds or thousands of specimens from many sites. Similarly, many absences will be nothing more than missing data, whereas absences in well-sampled sites may carry more meaning. These facts virtually call out for a probabilistic approach to the analysis of paleontological presence-absence data.

Here we describe a straightforward probabilistic model that contains parameters for the origination and extinction of taxa, for the ordering of the sites, and for the probabilities of errors (wrong zeros and wrong ones). Given the ordering of the sites, the origination and extinction parameters for a taxon specify the interval in which the taxon is assumed to be present. Any occurrence (a one in the matrix) of the taxon outside this interval is considered to be a false occurrence, and any nonoccurrence (a zero in the matrix) is considered to be a false occurrence as well. Given the parameters, the likelihood of the data depends on the number of false and true ones and zeros. The task we consider is to find parameter vectors that yield high likelihood, i.e., have a small number of false ones and zeros.

In more detail, our probabilistic model is as follows. Given a dataset with *N* sites and *M* taxa, we consider arbitrary orderings π of the sites. For each taxon *m,* we have the lifetime of the taxon, specified by the indices of the first and last occurrences *a_m_* and *b_m_* of taxon *m*. Additionally, we have parameters *c* for the probability of a false one (i.e., the presence of taxon *m* outside the bounds *a_m_* and *b_m_*) and *d* for the probability of a false zero (i.e., the absence of taxon *m* between the bounds *a_m_* and *b_m_*).

Denoting by θ the whole parameter vector (π, *a*, *b*, *c*, *d*), the likelihood of the data *X* has the form

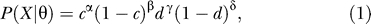
where α is the number of false ones, β is the number of correct zeros, γ is the number of false zeros, and δ is the number of correct ones.

We could, in principle, find a parameter vector θ that maximizes the likelihood of the data (maximum likelihood solution). This parameter vector would give a total order for the fossil sites, implying a probability of zero or one for a site pre-dating another. However, we know that the data contain pairs of sites from the same time periods. We are interested in finding pairs of sites for which the seriation is uncertain, i.e., the probability of one site pre-dating another is close to one-half.

Therefore, we work in the Bayesian framework, and find a sample of parameter vectors where the probability of a vector is proportional to its posterior probability. To this end, we use the Markov chain Monte Carlo (MCMC) method [28,29] to sample parameter combinations θ with probability proportional to their Bayesian posterior likelihood

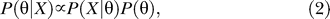
where *P*(θ) is the prior probability of the parameters θ.

MCMC methods yield samples from the posterior distribution of the parameters, and this makes it possible to study the space of the parameters in many different ways. For example, we can determine, for each pair of sites, the probability that one precedes the other. We can also estimate for the probability of false zeros and false ones and find for a particular observation in the data the probability that it is a false zero or a false one.

A further useful property of the model is that it is easy to incorporate additional information. For example, the model allows us to freeze the ordering of certain sites. That is, if we know that site *i* is definitely older than site *j*, we can restrict the MCMC method to accept only permutations that satisfy this constraint.

## Results

### Generated Data

We first ran the experiment on synthetically generated data, with known “true” ordering and probabilities of false zeros and ones for varying numbers of sites and taxa, shown in Table 1. The data were generated by forming *N* ordered sites and *M* taxa, assigning each taxon a lifespan (with median 0.18*N* sites) starting from a random position in the sitelist, and using the parameters *c* and *d* to produce false ones and false zeros.

**Table 1:**
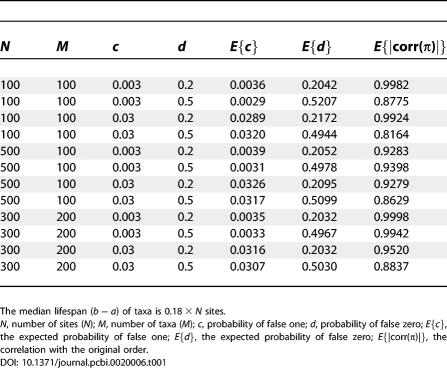
Results for Artificially Generated Datasets

The results on synthetic data show that the method quite accurately determines the parameters of the model: the expected values of *d* obtained from the MCMC simulation are close to the ones used in generating the data. The correlations between the original order and the MCMC orderings are also quite high; note that for high values of *c* and *d* it can be the case that some orderings different from the generating one fit the data better than the generating ordering.

### Cenozoic Large Land Mammal Data

In MCMC simulations, different runs can converge to separate regions in the parameter space. This is indeed what happens with the datasets on genera of Cenozoic large land mammals. We ran 100 MCMC chains over the datasets, and computed the variance in negative log-likelihood within the first chain, and then included all chains with the expected negative log-likelihood within one sigma of the best chain to our analysis.

The results are summarized in Table 2. The results show that the probability of a false one is quite low, whereas the probability of a false zero varies from 0.5 to 0.77, with the highest probabilities in the datasets where sites or genera with smaller occurrence frequencies are included. The correlation with the Mammal Neogene (MN) ordering and database age are also high.

**Table 2:**
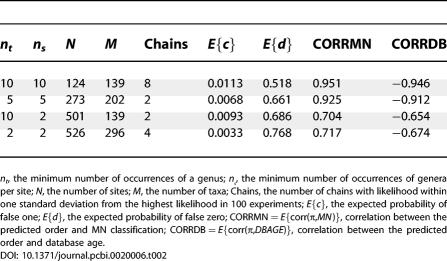
Results on the Large Mammal Dataset

**Figure 1:**
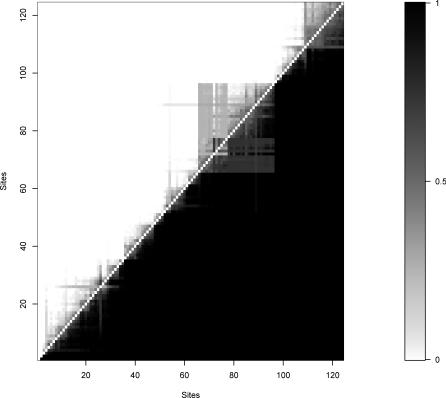
The Pair-Order Matrix *O_ij_ = P*(π(*i*) < π(*j*)) between Sites for Dataset *n_t_* = 10, *n_s_* = 10 from the Eight Chains with the Best Likelihood Black denotes probability one, and white denotes probability zero. For most pairs, the probability is close to zero or one, but some blocks of observations have many different orderings with high probability.

The probability that a site *i* occurs before site *j* can be estimated simply by counting in how many of the samples *i* precedes *j* in the ordering π. The results are shown in Figure 1. The ordering of the sites is in general quite well determined, but for some blocks of observations the ordering seems to be not fixed. Note that if two sites have exactly the same taxa, then the probability *P*(π(*i*) < π( *j*)) will be 0.5.

The pair-order matrices for all 100 chains are shown on our Web site (http://www.cis.hut.fi/projects/patdis/paleo). The chains outside the highest likelihood typically contain blocks of sites whose orders have been reversed; the MCMC method is fairly sensitive to initialization, and therefore running several chains with different initializations is useful.

**Figure 2:**
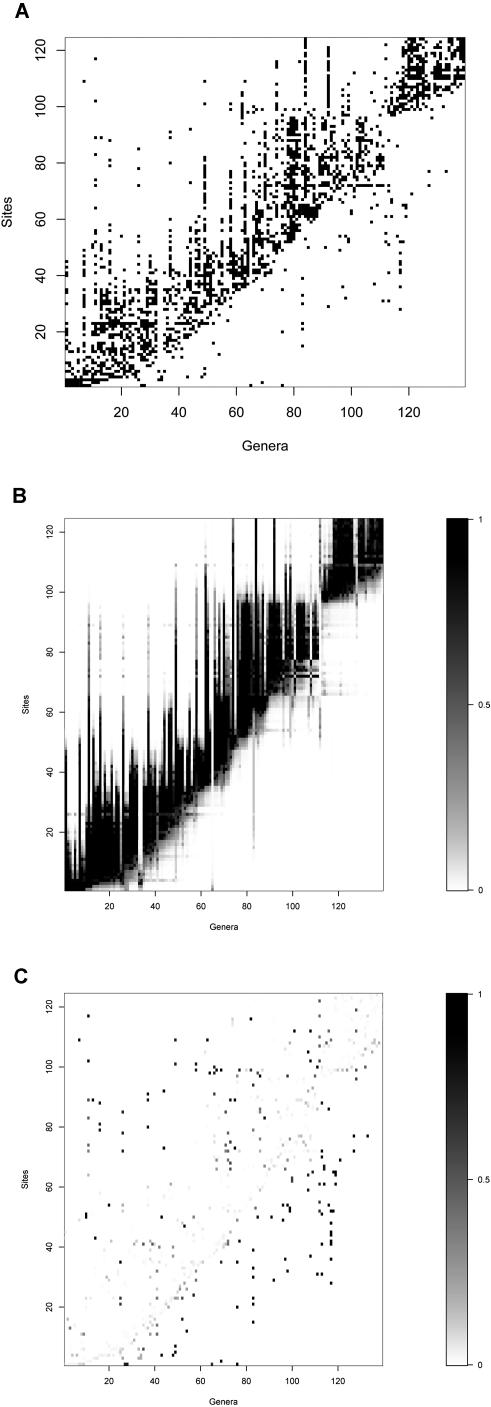
The Data Matrix for the Dataset with *n_t_* = 10 and *n_s_* = 10 The sites have been ordered by *E*{π(*n*)} and the genera by *E*{*a_m_*} (top). Probability that genus *m* is alive on site *n* in the dataset specified by *n_t_* = 10 and *n_s_* = 10 (middle). Probability that one is false (bottom). Black color denotes probability of one, and white probability of zero.

For the dataset specified by *n_t_* = 10 and *n_s_* = 10, the original data are shown in Figure 2A. The probability of a genus being alive at site *m* is shown in Figure 2B. Figure 2C shows for each one in the data the probability that it is a false one, i.e., that it falls outside the interval (*a_m_*, *b_m_*). We note that certain observations are quite strongly assumed to be false ones. The list of the strongest false ones is given on our Web site. Examination of the list shows that some of the observations that the model considers false ones are probably real errors in the data, while others represent true outliers or pertain to genera with unusual species or genera with artificially truncated distributions, as discussed below.

We further verified the detection of false zeros and ones by preparing two datasets, based on data parametrized by *n_t_* = 10 and *n_s_* = 10. For the first set, we selected 100 random ones, and flipped them to zero (false zeros). For the second set, we randomly selected 100 zeros, and changed them to ones (false ones). We then performed the analysis, and computed median probability for all zeros to be alive in the first dataset and median probability for all ones to be alive in the second datasets. The probabilities corresponding to 92 of 100 added false zeros was below the median, and 88 of 100 added false ones were above median. The differences are statistically significant when compared to the null hypothesis that the false zeros or ones are equally likely to end up above or below the median (Fisher Sign Test). The median probability that the (site,genus) pair an inserted false one is alive is 0.004, and the median probability that an inserted false zero is alive is 0.92.

We also tested a model where each taxon has its own *c* and *d* parameters for false one and false zero. The results of the MCMC runs were almost identical to the ones obtained for the model with one *c* and one *d* parameter (unpublished data).

## Discussion

We have described a probabilistic model for paleontological data and shown that MCMC methods can be used to obtain samples from the posterior distribution of the parameters. The parameters of the model have a natural interpretation, and the hard sites enable us to insert existing prior knowledge of the ordering in a natural way.

The task of finding the optimal ordering, or knowing for certain that a given ordering is optimal, is a very difficult problem. MCMC methods have the advantage of being able to explore various parts of the parameter space, but the issue of guaranteeing convergence of the sampling is always present in these methods. We have solved the problem of convergence by sampling 100 chains in parallel, and taking into account only the chains having the best log-likelihood. We have also checked that the pair-order matrices predicted by these best chains are consistent with each other. This way, we can state with reasonable confidence that our results are indeed an accurate description of the posterior distribution of the model. We also tested the method by adding false zeros and ones to the data randomly, and checking that they were identified correctly.

The results show that for generated data the method is able to reconstruct orderings and locate outliers with excellent accuracy. For the data on large late Cenozoic mammals, the results indicate a high level of agreement with existing orderings and correctly capture the basic feature of paleontological data that false absences are likely to be common and false presences rare.

For the past 40 years the main stratigraphic framework for the study of the Cenozoic land mammals from Europe has been the MN system [30–33]. The MN system rests on a complicated base of taxon appearances and associations that has been interpreted somewhat differently by different practitioners [33,34], who nevertheless usually agree on the MN assignment of most sites and virtually always agree on the sequence of MN reference localities. The probability pattern seen in Figure 1 is susceptible to a straightforward paleontological interpretation based on the ages assigned to them within the MN system and a general framework of climate change during the interval studied [35,36]. Starting from the lower left, the sites up to number 50 contain a sequence from the beginning of the Miocene at about 23 million years ago to the main faunal turnover event known in western Europe during this interval, the “Vallesian Crisis,” at about 10 million years ago. Sites 51–63 represent mostly the first million years of post-crisis time, while the large block between sites 63 and 99 represents the relatively stable latest Miocene from 8 million to 5 million years ago, known as the Turolian. A major faunal turnover event separates the Turolian from the Pliocene sites 100–117, and sites 118–124 represent the beginning of the last epoch of the Cenozoic, the Pleistocene. Of the two blocks of localities that the model cannot order well internally, the first and largest thus corresponds to an interval during which little change happened in the mammal faunas. In contrast, the second block, spanning the later Pliocene and the early Pleistocene, corresponds to a time of rapid climatic and faunal change, characterized by the increasingly prominent alternation of cold and warm intervals and, especially in Europe, the cyclic alternation of their attendant “cold” and “warm” mammal assemblages. As emphasized by [37,38], standard biochronology based on taxon ranges breaks down under conditions of cyclic change. The same is obviously true for our model, which assumes genus ranges to be continuous. However, the lack of resolution in this case can also be explained by the simple fact that all included localities are of very similar age, in particular since our data are at the genus level. It remains to be seen to what extent species-level data might resolve such blocks.

The structure just described is also evident in the patterns of Figure 2, where the observed and estimated taxon ranges of the ordered localities trace a pattern of three major faunal units separated by events where many genera go extinct and new, long-lived genera appear. Most marked of these is the turnover event associated with the Miocene–Pliocene transition at 5 million years ago, while the establishment of the two earlier faunal blocks appears more gradual. Figure 2 suggests that the model is especially skeptical of the early occurrences of genera, especially when their record is spotty, as for *Tapirus* (genus number 117 on the lower right).

Most genus occurrences considered by the model to be false are either genuine outliers in time and/or space or actual data errors. For example, of the ten cases at the head of the list for the *n_t_* = 10, *n_s_* = 10 dataset, *Plioviverrops* at Laugnac, *Thalassictis* at Wintershof-West, *Anancus* at Concud, and Dorn Dürkheim are true early occurrences, *Pliohyrax* at Pasalar and *Orycteropus* at Çandir are isolated early European occurrences of a genus of African origin, *Canis* at Concud is a similar occurrence of a genus dispersing from North America, *Plesiogulo* at Pasalar is a misidentification from a published preliminary list, while *Stephanorhinus* at Belometchetskaya and *Amphicyon* at Stavropol are probable curatorial errors in the NOW database.

Some apparent false occurrences reflect the biology of the animal in question. For example, the genus *Tapirus*, noted above for its spotty record, occurs eight times in the first 50 rows of probable errors. Several carnivore genera are also examples of genuinely rare but long-lived lineages, e.g., *Martes, Plesiogulo,* and *Mustela*. It thus appears that the model is a powerful tool to detect not only possible errors in the data but also genera with unusual distributional or ecological characteristics. This suggests that the method could also be of value for the study of the evolutionary dynamics of fossil communities [39,40]. It should be also noted that while the method is powerful and the results are useful, they should still be interpreted by an expert.

In [23], Halekoh and Vach considered an analogous problem in archeology: reconstruction of relative chronology of graves, based on the absence or presence information of finds. They construct a probabilistic model, similar in spirit to ours, but having more nuisance parameters. For example, where the appearance distribution of finds is unimodal and find-specific, i.e., the probability of false zero varies depending on the site and find. They solve the model with an MCMC algorithm, heavily tuned to address the convergence problems, and analyze the resulting modalities in detail. We solve the convergence problem by inserting strong prior information in terms of the hard sites (and fixing the direction of time), optimizing the sampling rules, and analyzing the results from 100 independent runs.

Like Alroy's [5] method, our model uses relative time, and ignores information about taphonomic regimes, sizes of collections, and biochronology; see [5] for further discussion. Computationally, Alroy's model has a likelihood function that is based on looking at the probability of not observing a conjunction (co-occurrence) of two taxa. For each taxon *i*, the model uses an individual parameter *κ_i_* (called the crypsis parameter), which corresponds to our Lazarus probability *c*. The probability that taxa *i* and *j* are not observed together in any site is in Alroy's model 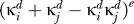 , where *e* is the number of sites for which the life spans of taxa *i* and *j* overlap and *d* is a parameter that typically is one. The taxon-specific probabilities of false ones and zeros can also be built into our model; the code available on our Web site provides for this. As mentioned, in the experiments this modification did not significantly change the results on real data. An interesting extension to the model would be to allow for temporal intervals for the sites.

One major difference between Alroy's model and ours is that we use MCMC methods to obtain a sample of the possible parameter values instead of looking for the maximum likelihood solution. This provides additional information about the robustness of the estimates. In particular, we tested our model by randomly adding false zeros and ones, and found that they were identified correctly.

## Materials and Methods

### The probabilistic model.

Formally, the *dataset* has *N* sites and *M* taxa, which are described with a 0–1 matrix *X_nm_*,where *n* ∈ {1,…,*N*} and *m* ∈ {1,…,*M*}. *X_nm_* = 1 signifies that remains of taxon *m* have been found from site *n*, while *X_nm_* = 0 means that no such discovery has been made. In the following, we describe in detail a probabilistic model that we propose has *generated* this dataset.

First, we assume that the sites appear in some temporal order, denoted by *permutation* π:{1,…,*N*}→{1,…,*N*}. We say that the site *i* is older than site *j*, if π(*i*) < π(*j*).

We further assume that there exists an ordering for all pairs of sites, i.e., for all *i* ≠ *j* we have either π(*i*) < π(*j*) or π(*j*) < π(*i*). Strictly speaking, this assumption is not always true. We may have two sites *i* and *j* that actually appeared more or less simultaneously. Should this be the case, our probabilistic model should predict *P*(π(*i*) < π(* j*)) ≈ *P*(π( *j*) < π(*i*)) ≈ 0.5; i.e., the probability of site *i* pre-dating site *j* should be about the same as site *j* pre-dating site *i*. We indeed find such sets of sites from our dataset.

We could take all *N*! permutations to be a priori equally likely. However, we know from various reasons outside of the dataset that some sites are, beyond any reasonable doubt, older than others. It would be foolish not to take this strong prior information into account when constructing the model. Formally, we introduce a set of pairs of sites, *hard orderings,* *H* = {(*h*_*i*1_, *h*_*h*2_)}_*i*∈{1, … , *N_H_*}_, order of which is known, i.e., π(*h*_*i*1_) < π(*h*_*i*2_). We denote by Π*_H_* a set of permutations that satisfy this order,


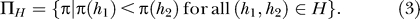


We assume that a priori all permutations in Π*_H_* are equally likely, and that permutations not appearing in Π*_H_* have a zero prior probability. The hard orderings make it possible to include existing information on the ordering of the sites into the model.

One should note that without hard ordering, i.e., when *H* is an empty set, our model is symmetric with respect to the time reversal. As a result, without hard orderings the posterior probability that site *i* pre-dates site *j* is always 0.5, making the pairwise time orderings of sites meaningless. Introducing hard orderings breaks this symmetry, after which the pairwise time orderings become non-trivial and meaningful. It should be noted that while the pairwise time ordering probability, *P*(π(*i*) < π(*j*)) = 0.5, is trivial without hard orderings, we could still get a meaningful measures of higher-order quantities, e.g., for the probability that the age of site *j* is between the ages of sites *i* and *k*, i.e., *P*(π(*i*) < π(*j*) < π(*k*) or π(*k*) < π(*j*) < π(*i*)).

One of the most basic properties of the taxa is that they originate and then go extinct at some later time. Therefore, for each taxon *m* we propose two parameters: the first signifying a site during which the taxon was first alive, *a_m_* ∈ [1, *N* + 1], and a site during which the taxon was first extinct, *b_m_* ∈ [1, *N* + 1]. We say that taxon *m* is *alive* on site *n* if *a_m_* ≤ π(*n*) < *b_m_*. Otherwise, the taxon is *dead*. We make the reasonable assumption that the taxa do not go extinct before they originate, i.e., *a_m_* ≤ *b_m_*. All pairs (*a_m_*, *b_m_*) satisfying this condition are a priori equally likely. *a_m_* = *b_m_* means the taxon *m* is not alive at any of the sites.

If our observations would be perfect, i.e., we would find samples of taxon if and only if it were alive (there would, e.g., be no Lasarus events), our time-ordered observation matrix *Y*, where *Y*_π(*n*)*m*_ = *X_nm_*, would consist of streaks of ones, signifying the presence of taxon (*Y_tm_* = 1 if *a_m_* ≤ *Y_tm_* < *b_m_*, *Y_tm_* = 0 otherwise). However, the observations are not perfect. Sometimes a taxon *m* is misidentified at site *n*, which may lead to false observation *X_nm_* = 1 even though the taxon should be dead at that particular site. On the other hand, it may happen for various reasons that taxon is not found from a particular site even though the taxon is alive, which will lead to false zero, *X_nm_* = 0.

We account for the imperfect observations by introducing two probabilities, the probability of false zero, *c* (we observe *X_nm_* = 1 even if the taxon is dead); and the probability of false zero, *d* (we observe *X_nm_* = 0 even if the taxon is alive). We assume log-uniform priors for *c* and *d* in the intervals 0.001 ≤ *c* ≤ 0.1 and 0.1 ≤ *d* ≤ 0.8, respectively. Summarizing, the parameters and priors of our model are given in Table 3. We denote all parameters collectively with

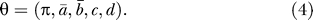
We also denote the prior of all parameters collectively by

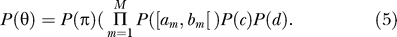
Given the parameters, we can finally specify the likelihood of the data,

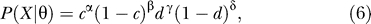
where α = ∑_*n*,*m*_ (1 − *e_nm_*)*X_nm_* is the number of false ones, β = ∑_*n*,*m*_ (1 − *e_nm_*) (1 − *X_nm_*) is the number of correct zeros, γ = ∑_*n*,*m*_ *e_nm_*(1 − *X_nm_*) is the number of false zeros, and δ = ∑_*n*,*m*_ *e_nm_X_nm_* is the number of correct ones, where we have used auxiliary Boolean parameter *e_nm_* to signify that the taxon is alive, i.e.,

**Table 3:**
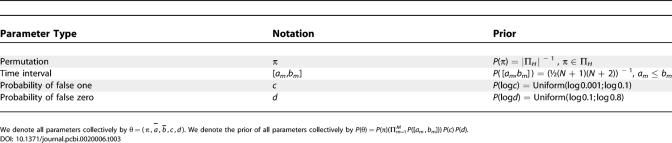
Parameters of Our Model, with Prior Distributions


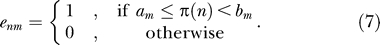


### Dataset.

We used a dataset of European late Cenozoic large land mammals derived from the NOW database (http://www.helsinki.fi/science/now) on 17 July 2003. We restricted the dataset to the Eurasian continent and islands in the Mediterranean Sea, excluding localities with greater than 60 degrees eastern longitude. We also restricted the dataset to the large mammal orders Primates, Creodonta, Carnivora, Perissodactyla, Artiodactyla, Proboscidea, Hyracoidea, and Tubulidentata. We considered three different kinds of age: database age, MN age, and geochronologic age. The MN system [30–33] is a classification of late Cenozoic into 18 classes.

For each locality, we calculated a database age as the mean of the minimum and maximum ages given in the original downloaded file. By MN age, we refer to the mean of the temporal boundaries of MN units according to the correlations given in [11]. This is given only for the subset of localities assigned in the database to a single MN unit or an interval expressed in MN units. For the MN 9 type locality Can Llobateres, we entered a regular MN 9 age in addition to the magnetostratigraphic age provided in the original NOW dataset. We also compiled a new age variable by copying all geochronologic (radiometric or magnetostratigraphic) age data in the original dataset to a separate variable. This new variable, referred to here as geochronologic age, was augmented by data taken from Appendix 2.1 of [11] (the main chronology used in the NOW dataset) and from recent updates for a set of Greek localities [41,42]. The dataset is available on our Web site (http://www.cis.hut.fi/projects/patdis/paleo).

We selected further data subsets as follows. First we selected the genera that occurred in at least *n_t_* in the original dataset; then we selected the sites in which at least *n_s_* such genera had been observed. We used the combinations (*n_t_*, *n_s_*) = (10,10), (5,5), (2,10) and (2,2). Note that, e.g., in the dataset with *n_t_* = 10 and *n_s_* = 10 several genera occur less than 10 times, as the selection on the number of genera is done first and then the sites are pruned.

We use the hard orderings of the sites, given by the MN reference sites,

π(Paulhiac)<             π(MontaiguleBlin)<

π(Laugnac)<             π(WintershofWest)<

π(LaRomieu)<            π(Pontlevoy)<

π(Sansan)<             π(LaGriveM)<

π(Can Llobateres I)<           π(Masía del Barbo)<

π(Crevillante 2)<           π(Los Mansuetos)<

π(Arquillo)<              π(Perpignan)<

π(Villafranca d′Asti (Arondelli))<     π(Saint Vallier)

Notice that all reference sites do not appear in all of the datasets, e.g., the dataset for *n_t_* = 10 and *n_s_* = 10 contains 11 of the 16 hard sites.

### The MCMC method.

Given the likelihood of Equation 6 and the prior of Equation 5, we can obtain the *posterior distribution* of the model parameters by applying the Bayes rule,

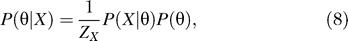
where the normalization factor is given by *Z_X_* = ∫ *P*(*X*|θ)*P*(θ)*d*θ.

We are interested in computing various interesting expectation values from the parameter distribution. If we know the posterior distribution, we can compute the expectations from integrand


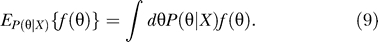


However, the analytic solution or integration of the posterior distribution is infeasible. Instead of solving the integral of Equation 9 directly, we use numerical integration, namely the MCMC method.

The MCMC algorithm allows us to draw samples from the posterior distribution, without the need for actually solving the Bayes equation. The MCMC algorithm gives us *T* samples of the parameters θ^*t*^, *t* ∈ {, … , *T*}, that satisfy *θ^t^* ~ *P*(*θ*|*X*) at the limit of large *T*. Given the samples, we can then approximate the integral of Equation 9 by


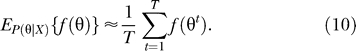


The Markov chain in the name of the MCMC algorithm comes from the fact that a posterior sample is a stochastic function of the previous posterior sample and the data, *θ^t+1^* ~ *g*(*θ^t^*, *X*). Thus, the MCMC samples form a *chain* in parameter space: θ^1^, θ^2^, …, θ^T^. The consecutive samples in the chain are not independent. If the chain is too short (*T* is small), one chain can effectively cover only a small fraction of the full probability mass.

We first initialize each chain with random values, as follows:

Initial permutation is drawn from the prior, π^1^ ~ *P*(π). The initial ordering of the sites is thus totally random, with the restriction that the “hard” orderings given by the set Π*_H_* are enforced.

The initial intervals are set to smallest intervals that have no false ones 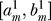 , given the initial permutation π^1^.

The probabilities of false ones and zeros are initialized to *c* ^1^ = 0.01 and *d* ^1^ = 0.3.

After the initialization, we run the chain for the *T_B_* = 10,000 step *burn-in period*. The posterior samples drawn during the burn-in period are ignored. The purpose of the burn-in period is to initialize the chain and to bring it to the area of large probability mass in the posterior distribution. We use the final state of the burn-in period, θ*^T_B_^* to initialize the actual chain that we use to calculate the expectations. We run the chain for *T′* = 10,000 iterations and save every tenth sample, resulting in *T* = 1,000 samples per chain. We then use these stored samples to calculate the desired expectations.

In MCMC methods, the question of convergence always arises. The parameter space may have areas of large probability mass that are separate in the sense that it is very unlikely that a chain jumps from one of these regions to another. It is possible that a chain ends up in these regions, resulting in inaccurate expectations due to the fact that the integration effectively takes only a small subset of the posterior mass into account. Indeed, efficient sampling of the full parameter space is in a general case a very difficult problem. The problems of finding the maximum likelihood solution for these types of problems are typically NP-hard [43,44]. Therefore, the best we can do is to build our algorithm so that the chains of large probability mass can be identified with a reasonable confidence (see [29] for review of convergence criteria).

We proceed in two steps: first, we run 100 chains in parallel, and compute the expected log-likelihood, *E*{*logP*(*X*|θ)}, of the data for each of the chains. We then compute the standard deviation of the log-likelihoods of the chains and then use only the chains with the expected log-likelihood within one standard deviation of the best chain into account. For example, with the dataset with *n_t_* = 10 and *n_s_* = 10 we end up selecting eight of the original 100 chains.

If the predictions given by the chains having the high log-likelihood are consistent with each other, we can conclude that the chains have converged well and that the results are reliable. However, if the predictions given by chains would differ, we could conclude that the chains have converged to separate regions in the parameter space and we also get an estimate for the error due to bad convergence.

Specifically, assume that we analyze *K* chains, each with unique initialization and *T* samples 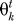 , after the burn-in period. The individual chains produce expectation of a function *f*(θ) of the parameters θ

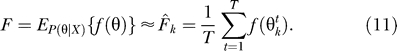
The expectation given by all *K* chains is given by


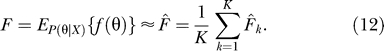


If the expectations *F̂_k_* produced by individual chains are similar (using some suitable distance measure *d* ^2^) to the combined result, *F̂*, i.e., *d* ^2^(*F̂_k_*, *F̂*) is small for all *k*, then we can have some confidence on the approximation *F̂* of the expectation *F*. Indeed, the distance measure *d* ^2^(*F̂_k_*, *F̂*) gives an approximation of the error of the prediction *F̂* to the true expectation *F*, *d* ^2^(*F*,*F̂*).

We use the expectations of pair-order probabilities and their Hellinger divergences as *d* ^2^ to measure the similarity of the chains (see below).

The actual sampling rules are given below. In our implementation run of one chain over the dataset with *n_t_* = 10 and *n_s_* = 10 (*N* = 124, *M* = 139), we used 10,000 burn-in iterations and equal number of actual sampling iterations. One run takes about 8 min on a low-end desktop (Mac mini with a 1.42 GHz G4 processor). Our C implementation is available for download from our Web site at http://www.cis.hut.fi/projects/patdis/paleo. The time and memory requirements of one sampling iteration scale linearly with the size of the data matrix, the time and memory usage being *O*(*NM*).

### Sampling rules.

In this section, we describe the details of the sampling methods we have used. We use *Y* to denote the time-ordered matrix of observations, *Y*_π(*i*)*j*_ = *X_ij_*; and π^−1^ to denote inverse permutation, defined by π ^−1^(π(*i*)) = *i*.

### Permutations.

The permutation of order, π, is most difficult to sample efficiently. To compensate for this difficulty, we have constructed four sampling iterations for the permutations, which we iterate five times for each MCMC step.

The first sampling method consists of moving site *n* from time π(*n*) to a new time *i*, simultaneously moving all sites and interval limits accordingly. We first define the following auxiliary methods, TOYOUNGER and TOOLDER:

TOYOUNGER(*i*, *j*):

Let *aux* ← π(*i*).

For *k* in *i*,…,*j* − 1:

Let π(*k*) ← π(*k* + 1).

Let π(*j*) ← *aux*.

Let *a_m_* ← *a_m_* − 1 for all *a_m_* ∈ ]*i*,*j* + 1].

Let *b_m_* ← *b_m_* − 1 for all *b_m_* ∈ ]*i*,*j* + 1].

TOOLDER(*i*, *j*):

Let *aux* ← π(*j*).

For *k* in *j*,…,*i* + 1:

Let π(*k*) ← π(*k* − 1).

Let π(*i*) ← *aux*.

Let *a_m_* ← *a_m_* + 1 for all *a_m_* ∈ [*i*,*j* + 1[.

Let *b_m_* ← *b_m_* + 1 for all *b_m_* ∈ [*i*,*j* + 1[.

The actual MCMC step consists of moving a site *n* with time index *i* = π(*n*) to time index *j*, simultaneously shifting all intervening sites and limits by one accordingly:

MOVEONESITE(*i*, *j*):

If *i* < *j*:

TOYOUNGER(*i*, *j*)

Else if *j* < *i*:

TOOLDER(*j*, *i*)

The sample is then taken by first selecting a random pair of indices, *i* and *j*, and then excuting the step MOVEONESITE(*i*, *j*) with Metropolis-Hastings probability (“SAMPLEPI1”).

The second part of sampling of π consists of selecting an interval [*i*,*j*[, and reversing the order of sites within the interval. In pseudocode, the transformation reads, where we have used π^−1^(*i*:*j*) to denote a vector of site indices, π^−1^(*i*:*j*) = (π^−1^(*i*),…,π^−1^(*j*)), and reverse(□) to denote the reverse of vector □.

REVERSE1(*i*, *j*, *B*):

Let π^−1^(*i*:*j*) ← reverse(π^− 1^(*i*:*j*)).

Let *a_m_* ← *i* + *j* + 1 − *a_m_* for all *a_m_* ∈ *B*.

Let *b_m_* ← *i* + *j* + 1 − *b_m_* for all *b_m_* ∈ *B*.

Swap *a_m_* and *b_m_*, if *a_m_* ∈ *B* and *b_m_* ∈ *B*.

The sample is then taken by first selecting a random interval *i*,*j* ∈ {1,…,*N* + 1}, *i* < *j* and by selecting randomly one of the following sets: *B* = [*i*,*j* + 1[, *B* = [*i*,*j* + 1], *B* = ]*i*,*j* + 1[, *B* = ]*i*,*j* + 1]. The step REVERSE1(*i*, *j*, *B*) is then executed with the Metropolis-Hastings probability and denoted by “SAMPLEPI2”.

The third sampling rule for π, REVERSE2(*i*, *j*, *B*), is similar to REVERSE1, with the exception that only the order of non-hard sites is reversed, denoted by “SAMPLEPI3”. The hard sites are left untouched.

The fourth sampling rule consists of swapping neighboring sites, i.e.,

REVERSE1(*i*, *i* + 1), denoted by “SAMPLEPI4”.

### Other parameters.

After sampling for the permutation, we proceed to sample the parameters *c* (probability of false one) and *d* (probability of false zero). This sampling is done with the aid of the Metropolis-Hastings algorithm. We propose an update to log*c* by sampling the proposal from the normal distribution, log*c′* ~ *N*(log*c*,0.15). We accept the proposal with the Metropolis-Hastings probability *min*(1,*P*(*X*|θ′)/*P*(*X*|θ)), where θ denotes the original parameters and θ′ the proposed parameters—in this case, the parameters with a new value of *c*. The sampling for the probability of false zero, *d*, proceeds analogously.

To sample for the parameters *a_m_* (site where the taxon is first alive), for each *m* ∈ {1,…,*M*}, we calculate the relative likelihood of the data for all *a_m_* ∈ [0,*b_m_*]. This calculation can be done efficiently in *O*(*N*) steps. We then normalize these likelihoods to unity and sample new *a_m_* from Multinomial(*p*;*b_m_* + 1). The sampling for *b_m_* proceeds analogously.

Summarizing, one sampling iteration consists of one sampling of *c*, *d*, *a*, *b* and SAMPLEPI4; and five iterations of SAMPLEPI1, SAMPLEPI2, and SAMPLEPI3. The actual sampling consists of 10,000 of these iterations, of which every tenth sample is stored for use in analysis. The C-implementation of the sampling program is available for download at http://www.cis.hut.fi/projects/patdis/paleo.

### Experimental setup.

We can visualize a MCMC chain with a *pair-order matrix,* defined by

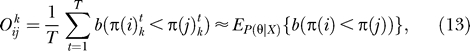
where the indices *i* and *j* denote sites, *k* a particular chain and *b*(□) is a boolean function that equals one when □ is satisfied, and zero otherwise. Thus 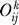 equals the probability that the site *i* pre-dates the site *j*. Furthermore, the pair-order matrix satisfies 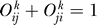 . We can also compare two chains by defining a distance measure using the averaged Hellinger divergences [45] over two pair-order matrices, i.e.,

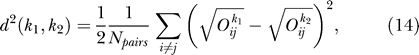
where 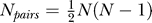 . The distance measure satisfies *d* ^2^(*k*_1_, *k*_2_) ∈ [0, 1] and it is equal to zero only if the pair-order matrices are equal. The average Hellinger distance between the pair-order matrices of the eight chains used in the analysis of the dataset with *n_t_* = 10 and *n_s_* = 10 is 0.010.

## Abbreviations

MCMC: Markov chain Monte Carlo
MN: Mammal Neogene
